# Combination photoimmunotherapy with monoclonal antibodies recognizing different epitopes of human epidermal growth factor receptor 2: an assessment of phototherapeutic effect based on fluorescence molecular imaging

**DOI:** 10.18632/oncotarget.7490

**Published:** 2016-02-19

**Authors:** Kimihiro Ito, Makoto Mitsunaga, Takashi Nishimura, Hisataka Kobayashi, Hisao Tajiri

**Affiliations:** ^1^ Division of Gastroenterology and Hepatology, Department of Internal Medicine, The Jikei University School of Medicine, Minato, Tokyo 105-8461, Japan; ^2^ Molecular Imaging Program, Center for Cancer Research, National Cancer Institute, Bethesda, MD 20892-1088, USA

**Keywords:** molecular targeted therapy, photoimmunotherapy, HER2, trastuzumab, pertuzumab

## Abstract

Photoimmunotherapy is a new class of molecular targeted cancer therapy based on a monoclonal antibody (mAb) conjugated to a photosensitizer and irradiation with near-infrared (NIR) light for both imaging and therapy. Here, we sought to determine the feasibility of combining photoimmunotherapy using conjugates of human epidermal growth factor receptor 2 (HER2)-specific mAb-photosensitizer IR700, trastuzumab-IR700 and pertuzumab-IR700. HER2-expressing and non-expressing cells were treated with mAb-IR700 conjugates and irradiated with NIR light. Fluorescence imaging and cytotoxic effects were examined in cultured HER2-expressng cancer cell lines and in a mouse tumor xenograft model. Trastuzumab-IR700 and pertuzumab-IR700 could specifically bind to HER2 without competing, and the combination treatment of both agents yielded stronger HER2-specific IR700 fluorescence signals than with the treatment with either agent singly. A cytotoxicity assay showed that the combination treatment of both trastuzumab-IR700 and pertuzumab-IR700 followed by NIR light irradiation induced stronger cytotoxic effect than with treatment of either agent plus NIR light irradiation. Furthermore, the phototoxic and cytotoxic effects of mAb depended on HER2-specific IR700 signal intensities. Consistent with *in vitro* studies, in xenograft tumor models also, IR700 fluorescence imaging-guided NIR light irradiation after the combination treatment of trastuzumab-IR700 and pertuzumab-IR700 led to stronger antitumor effects than by treatment with either agent followed by NIR light irradiation. In conclusion, fluorescence molecular imaging can facilitate the assessment of treatment outcomes of molecular targeted photoimmunotherapy, which holds great potential in facilitating better outcomes in cancer patients.

## INTRODUCTION

Human epidermal growth factor receptor 2 (HER2) is a member of the epidermal growth factor receptor family, which regulates cell proliferation, differentiation, and apoptosis through signal transduction by forming homodimers or heterodimers [1]. HER2 is commonly expressed on the membrane of various types of cancer cells, and its overexpression is associated with tumor malignancy [2]. Trastuzumab, a humanized anti-HER2 monoclonal antibody (mAb) directed against domain IV of HER2, manifests its antitumor activity by inducing antibody-dependent cellular cytotoxicity, inhibiting ligand-independent HER2 signaling, blocking active formation of HER2, and preventing the cleavage of HER2 [3, 4]. Thus, trastuzumab is widely used for treating HER2-expressing cancers as a single agent or in combination with chemotherapy [5, 6]. Pertuzumab, a humanized anti-HER2 mAb directed against domain II of HER2, has been recently developed, and it evinces its antitumor activity by inhibiting the dimerization of HER2 with other HER family proteins, thereby preventing HER2-mediated signaling [7]. In preclinical and clinical reports, the combined treatment of these 2 anti-HER2 mAbs has been found to induce a stronger antitumor effect on HER2-expressing cancers than with treatment with either agent singly, owing to the different mechanisms of HER2 signal inhibition [8–10].

Photoimmunotherapy (PIT) is a new class of molecular-targeted cancer therapy based on a mAb conjugated to a photosensitizing phthalocyanine dye, IR700, followed by near-infrared (NIR) light irradiation under the guidance of molecular-targeted fluorescence imaging. The mAb-IR700 conjugate binds the target molecules on the cell membrane and rapidly induces cellular necrosis and rupture of the membrane by the photo-activated IR700 after NIR light exposure, without significant cytotoxic effects towards normal cells, to which mAb-IR700 is not bound [11–13].

PIT has demonstrated HER2-targeted phototoxicity in various HER2-expressing cancer mouse models using trastuzumab-IR700 conjugates [14–17]. Moreover, a recent study demonstrated that the more mAb-IR700 binding to cells, the stronger the PIT effect induced at the same dose of NIR light irradiation [18]. Here, we hypothesized that the combination treatment of the 2 different mAb-IR700 types, namely, trasutumab-IR700 (Tra-IR700) and pertuzumab-IR700 (Per-IR700), which bind to different epitopes of HER2, achieves higher IR700 binding and results in a stronger PIT effect compared to PIT mediated by treatment with either mAb-IR700 alone.

## RESULTS

### HER2 expression *in vitro*

**Figure 1:**
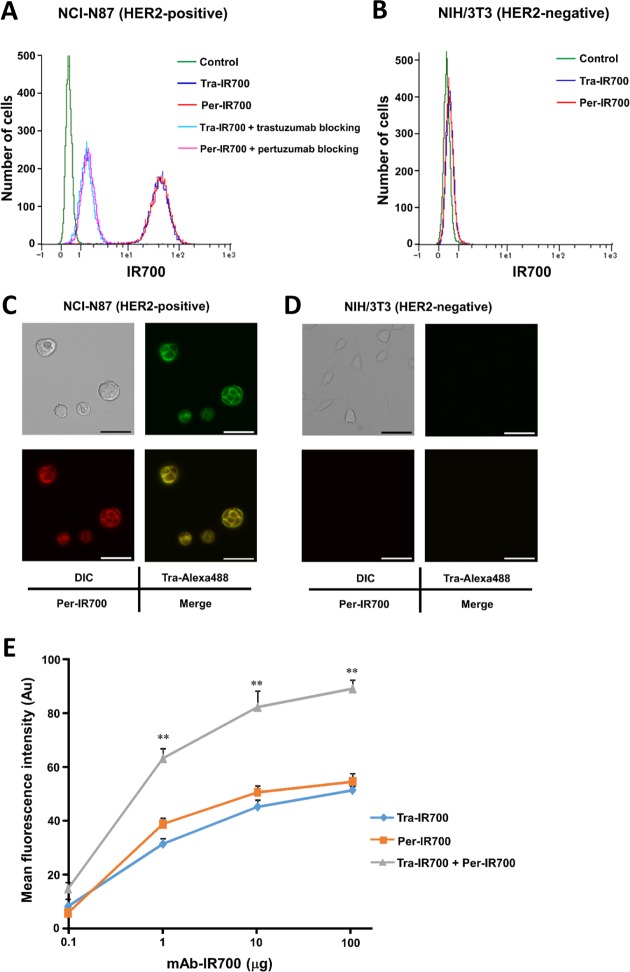
Human epidermal growth factor receptor 2 (HER2) expression in NCI-N87 and NIH/3T3 cells *in vitro* (**A, B**) Flow cytometry analysis revealed strong HER2-specific binding of Tra-IR700 and Per-IR700 in NCI-N87 cells but not in NIH/3T3 cells. Specific binding was demonstrated by excess unconjugated antibody blocking. (**C, D**) Fluorescence microscopy showed HER2-specific localization of Tra-Alexa488 and Per-IR700 in NCI-N87 cells but not in NIH/3T3 cells. These fluorescence signals were co-localized on the cell surface. DIC: differential interference contrast. Scale bar = 50 μm. (**E**) Flow cytometry analysis showed that NCI-N87 cells produced significantly stronger IR700 signals when treated with both Tra-IR700 and Per-IR700 compared to treatment with either agent alone. Data are presented as means ± SEM (*n* = 3, **P < 0.01 vs. either agent at the same concentration, Student's *t*-test).

After 3-h incubation with either Tra-IR700 or Per-IR700, HER2-expressing NCI-N87 cells showed strong IR700 fluorescence (Figure 1A). The ratios of the mean fluorescence intensity (MFI) compared to the isotype control were 119.2.9 ± 5.9 for Tra-IR700 and 124.4 ± 6.6 for Per-IR700 respectively (means ± SEM, *n* = 3). These signals were almost completely blocked by adding excess unconjugated trastuzumab or pertuzumab. The ratios of the MFI compared to the isotype control were 4.4 ± 0.4 for Tra-IR700 with trastuzumab blocking and 4.5 ± 0.3 for Per-IR700 with pertuzumab blocking (means ± SEM, *n* = 3), suggesting HER2-specific binding of Tra-IR700 and Per-IR700. In contrast, Tra-IR700 signal was not blocked by excess unconjugated pertuzumab, and Per-IR700 signal was not blocked by excess unconjugated trastuzumab, suggesting epitope specificity of trastuzumab and pertuzumab (Supplementary Figure S1). There was no significant difference in signals between Tra-IR700 or Per-IR700 treatment and the isotype control treatment for HER2-negative NIH/3T3 cells (Figure 1B). To detect HER2-specific localization of trastuzumab and pertuzumab, fluorescence microscopy was performed after 3-h incubation with Tra-Alexa488 and Per-IR700. Alexa488 and IR700 fluorescence were detected at the same locations, predominantly on the cell surface of NCI-N87 cells, while NIH/3T3 cells did not show any detectable fluorescence for Alexa488 or IR700 under the same camera conditions (Figure 1C, 1D).

### Enhanced HER2-speicfic IR700 fluorescence signals with combined treatment of Tra-IR700 and Per-IR700 *in vitro*

When cells were treated with Tra-IR700 or Per-IR700 alone, IR700 signals increased in a dose-dependent manner but were almost saturated by treatment with 10 μg of the respective single agent. Interestingly, when cells were treated with both Tra-IR700 and Per-IR700, IR700 signals increased in a dose-dependent manner and exceeded the saturation level of single-agent treatment, even at a dose as low as 1 μg each of Tra-IR700 and Per-IR700 (Figure 1E).

### HER2-specific IR700 fluorescence signal intensity indicated that Tra-IR700- and Per-IR700-mediated PIT enhances the phototoxic effect

**Figure 2:**
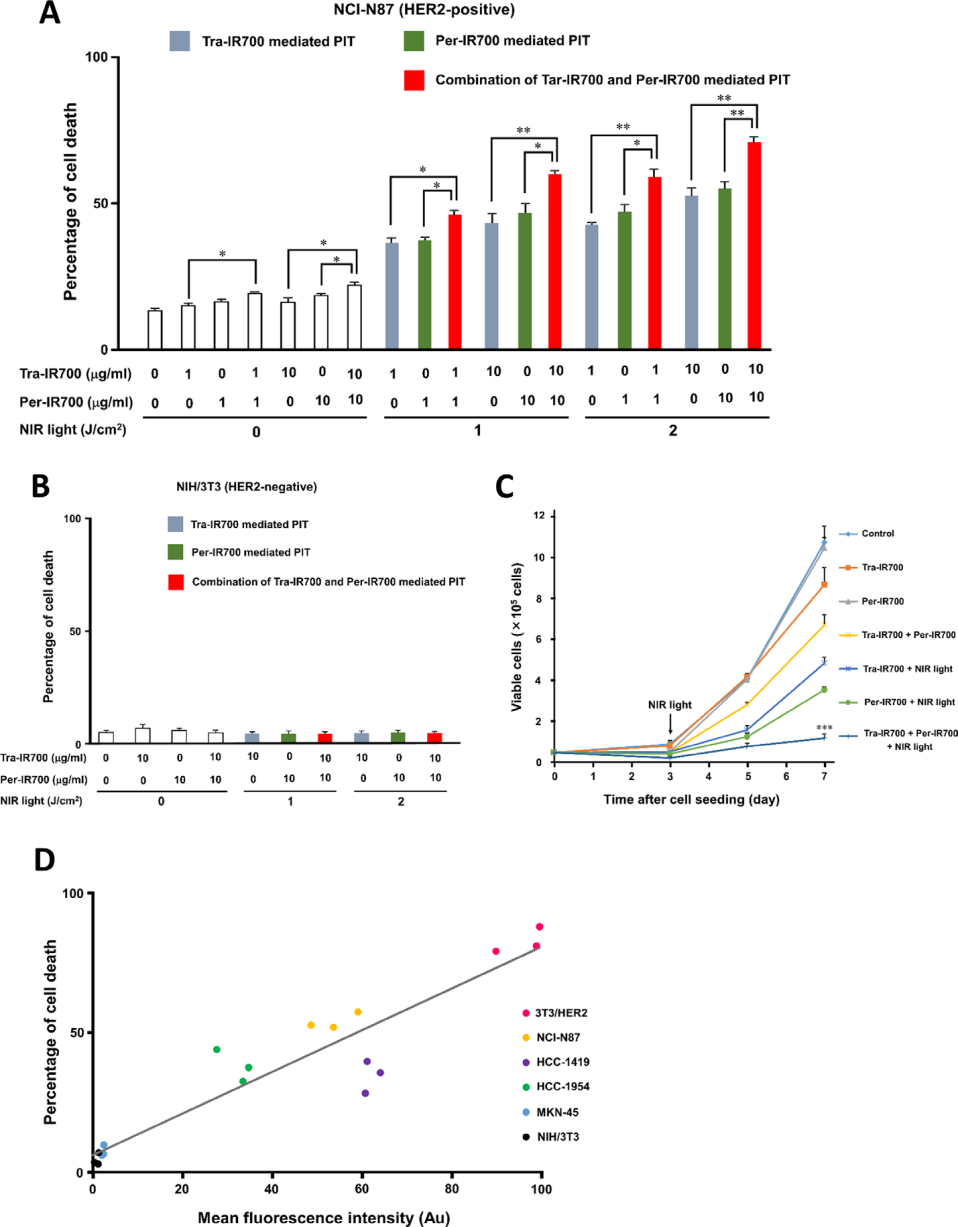
Trastuzumab-IR700- and pertuzumab-IR700-mediated photoimmunotherapy leads to enhanced phototoxic effect compared to photoimmunotherapy using either agent *in vitro* (**A**) LIVE/DEAD assay in NCI-N87 cells showed that the percentage of cell death increased significantly with combination treatment with Tra-IR700 and Per-IR700 followed by NIR light compared to treatment with either agent singly followed by NIR light. Data are presented as means ± SEM (*n* = 3, *P < 0.5, **P < 0.05, Student's *t*-test). (**B**) No cytotoxicity associated with mAb-IR700 treatment or NIR light irradiation was found in NIH/3T3 cells. (**C**) A trypan blue dye exclusion assay showed significant growth inhibition in the cells treated with 10 μg/ml each of Tra-IR700 and Per-IR700 followed by 2 J/cm^2^ NIR light irradiation, compared to treatment with 10 μg/ml of either agent followed by 2 J/cm^2^ NIR light irradiation (*n* = 3, ***P < 0.001 vs. Tra-IR700 with NIR light, ***P < 0.001 vs. Per-IR700 with NIR light, Student's *t*-test). (**D**) There was a positive correlation between the MFI of HER2-specific IR700 signals from the cells treated with Tra-IR700 and the percentage of cell death in response to Tra-IR700-mediated PIT (*P* < 0.0001, *R*^2^ = 0.87, Pearson's correlation coefficient).

The percentage of cell death by trastuzumab or pertuzumab treatment (10 μg/ml) was slightly higher than that in the control NCI-N87 cells but was significantly lower than that by the combination treatment. In addition, there was no significant difference in cytotoxicity between trastuzumab and Tra-IR700, between pertuzumab and Per-IR700, or between the combination of trastuzumab and pertuzumab and combination of Tra-IR700 and Per-IR700 treatment (Supplementary Figure S2). When cells were treated with either Tra-IR700 or Per-IR700 followed by NIR light irradiation, the percentage of cell death increased relative to the dose of mAb-IR700 and NIR light dose (Figure 2A). In addition, cell death significantly increased when cells were treated with both Tra-IR700 and Per-IR700 followed by NIR light irradiation, compared to single-agent treatment followed by NIR light irradiation (Figure 2A). Furthermore, a similar cytotoxic effect was found when cells were treated with 1 μg/ml each of Tra-IR700 and Per-IR700 together followed by NIR light irradiation compared to 10 μg/ml of either agent alone followed by NIR light. Importantly, no cytotoxicity associated with mAb-IR700 treatment or NIR light irradiation was observed in HER2-negative NIH/3T3 cells (Figure 2B). The trypan blue dye exclusion assay, which reflects long-term cytotoxic effect in response to PIT, showed significant growth inhibition in the cells treated with both Tra-IR700 and Per-IR700 followed by NIR light irradiation compared to treatment with either agent alone followed by the irradiation (Figure 2C).

We next examined the correlation between HER2-specific IR700 signals and phototoxicity in response to PIT by using cell lines expressing different HER2 expression levels. The MFIs were 96.1 ± 3.1, 53.7 ± 3.0, 61.9 ± 1.0, 31.9 ± 2.2, 2.3 ± 0.1, and 0.96 ± 0.3 for 3T3/HER2, NCI-N87, HCC-1419, HCC-1954, MKN-45, and NIH/3T3, respectively (arbitrary unit, means ± SEM, *n* = 3). A positive correlation was seen between the MFIs of IR700 signals from the cells treated with Tra-IR700 and the percentage of cell death in response to Tra-IR700-mediated PIT (*P* < 0.0001, *R*^2^ = 0.87, Pearson's correlation coefficient) (Figure 2D).

### *In vivo* biodistribution of Tra-IR700 and Per-IR700

**Figure 3:**
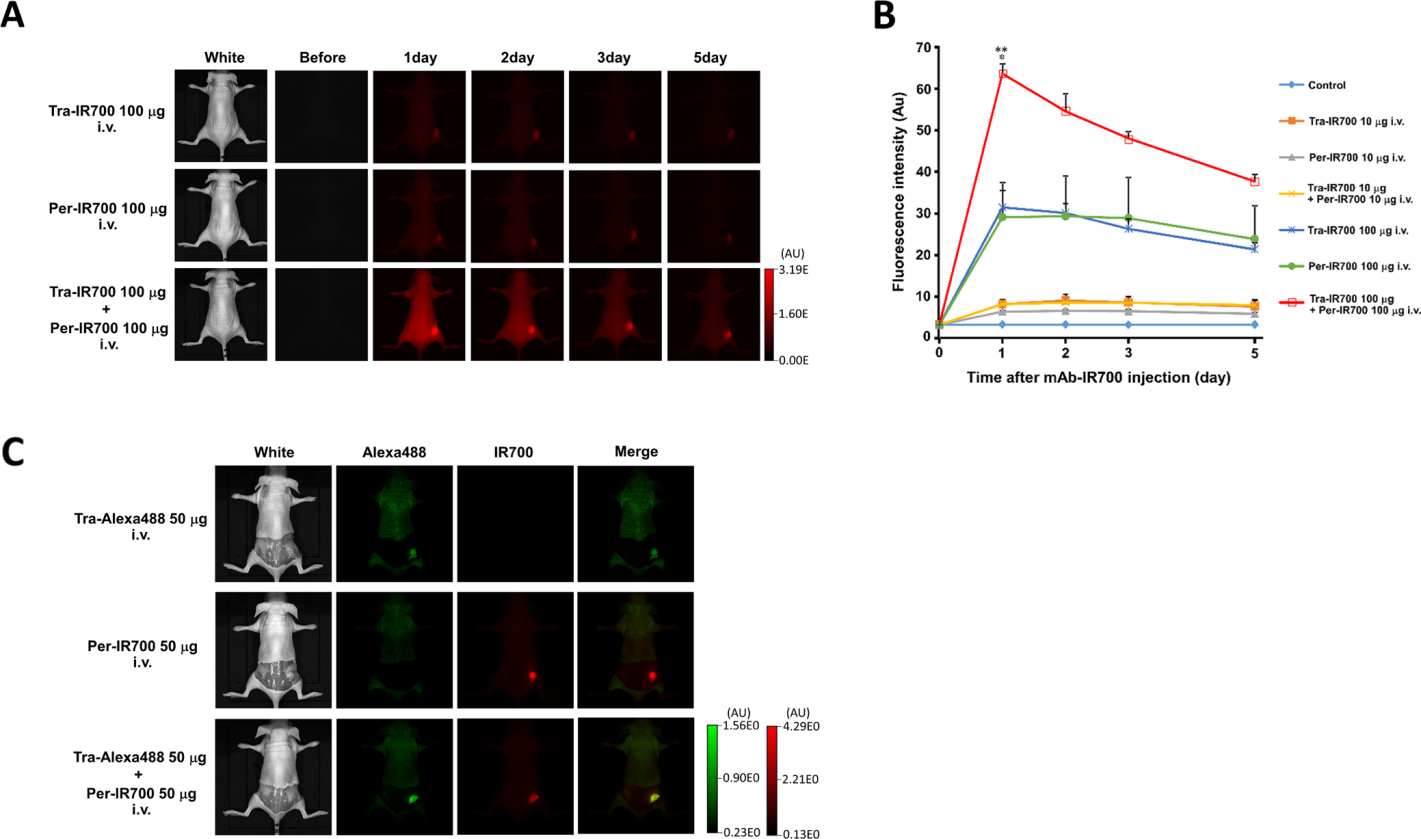
*In vivo* biodistribution of trastuzumab-IR700 and pertuzumab-IR700 (**A, B**) NCI-N87 tumor xenografts were visualized with IR700 fluorescence after intravenous injection of Tra-IR700 and/or Per-IR700. Stronger IR700 signals were observed 1 day after the injection of 100 μg each of Tra-IR700 and Per-IR700 than injection of either agent alone. Data are presented as means ± SEM (*n* = 3, **P < 0.01: 100 μg of Tra-IR700 and 100 μg of Per-IR700 i.v. vs. 100 μg of Tra-IR700 i.v., *P < 0.05: 100 μg of Tra-IR700 and 100 μg of Per-IR700 i.v. vs. 100 μg of Per-IR700 i.v., Student's *t*-test). (**C**) NCI-N87 tumors (right dorsum) were specifically visualized with Tra-Alexa488 and Per-IR700 fluorescence, while NIH/3T3 tumors (left dorsum) did not show detectable fluorescence signals (*n* = 3).

To examine the biodistribution of Tra-IR700 and Per-IR700 in the xenograft tumor model, serial fluorescence images were obtained before and after injection of Tra-IR700 and/or Per-IR700. NCI-N87 tumors were visualized with IR700 fluorescence 1 day after the injection, and fluorescence signal intensity decreased gradually thereafter (Figure 3A, 3B). No other IR700 localization was found except for the NCI-N87 tumors. Quantitative analysis of IR700 fluorescence in NCI-N87 tumors showed higher signal intensity after injection of both 100 μg of Tra-IR700 and 100 μg of Per-IR700 than injection of the same amount of either agent alone (*n* = 3, 1 day after injection; **P = 0.0023: 100 μg of Tra-IR700 and 100 μg of Per-IR700 i.v. vs. 100 μg of Tra-IR700 i.v., *P = 0.016: 100 μg of Tra-IR700 and 100 μg of Per-IR700 i.v. vs. 100 μg of Per-IR700 i.v.) (Figure 3B). A similar additive effect was found when mice were treated with both 10 μg of Tra-IR700 and 10 μg of Per-IR700 compared to treatment with either agent alone. However, the IR700 signal intensity was much lower than in mice treated with 100 μg each of either agent. Furthermore, to detect HER2-specific localization of trastuzumab and pertuzumab in target tumors, both NCI-N87 and NIH/3T3 tumor-bearing mice were created, and fluorescence images were obtained after injection of both Tra-Alexa488 and Per-IR700. Since the fluorescence signal of Alexa488 could not detect tumors even in the xenograft tumor mice in our *in vivo* imaging system, the dorsum skin was removed for acquiring Tra-Alexa488 signals (Supplementary Figure S3). Image analysis showed that NCI-N87 tumors were specifically visualized with both Alexa488 and IR700 fluorescence in the same region of NCI-N87 tumor, and there was no crosstalk between Alexa488 and IR700 fluorescence, while NIH/3T3 tumors did not show apparent fluorescence signals (Figure 3C).

### Combination treatment of Tra-IR700- and Per-IR700-mediated PIT leads to a stronger antitumor effect than by treatment with either agent alone

**Figure 4:**
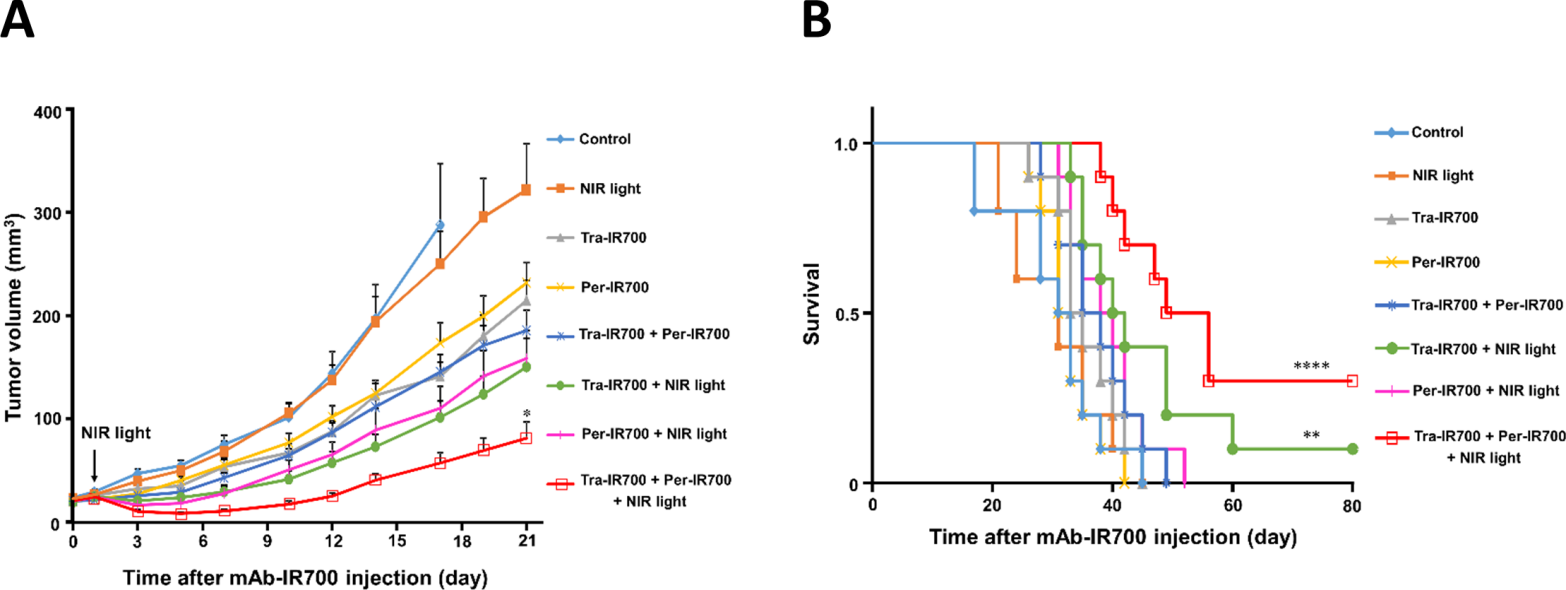
Stronger antitumor effect by combination treatment of trastuzumab-IR700 and pertuzumab-IR700 with NIR light irradiation *in vivo* (**A**) Combination treatment with both Tra-IR700 and Per-IR700, together with NIR light irradiation, led to a stronger antitumor effect than treatment with single agents and NIR light irradiation; none of the treatments had any apparent side effects. Data are presented as means ± SEM (*n* = 10 in each group, *P < 0.05: Tra-IR700 and Per-IR700 with NIR light vs. Tra-IR700 or Per-IR700 with NIR light; Mann–Whitney *U* test). (**B**) Combination treatment of Tra-IR700 and Per-IR700 with NIR light radiation led to prolonged survival compared to the control (*n* = 10 in each group, ****P < 0.0001, **P < 0.01 vs. control; log-rank test).

Tumor xenografts reached 20 mm^3^ in volume approximately 7 days after subcutaneous injection of NCI-N87 cells. Tumors were irradiated with a single dose of NIR light on day 1 after intravenous injection of mAb-IR700 under the guidance of IR700 fluorescence, because the highest IR700 accumulation was observed at that time point. As compared with the control group, long-term growth inhibition was observed in the groups of Tra-IR700, Per-IR700, and Tra-IR700 plus Per-IR700 (Figure 4A). In addition, significant growth inhibition was observed in the groups of Tra-IR700 with NIR light, Per-IR700 with NIR light, and both Tra-IR700 and Per-IR700 with NIR light, starting at a few days after NIR light irradiation. Moreover, combination treatment of both Tra-IR700 and Per-IR700 with NIR light leads to a stronger antitumor effect than by treatment with either single agent with NIR light, without any apparent side effects (*n* = 10 in each group, 21 days after mAb-IR700 injection; *P = 0.0339: Tra-IR700 and Per-IR700 with NIR light vs. Tra-IR700 with NIR light, *P = 0.0185: Tra-IR700 and Per-IR700 with NIR light vs. Per-IR700 with NIR light; Mann–Whitney *U* test) (Figure 4A). In addition, survival was prolonged significantly when mice were treated with Tra-IR700 and Per-IR700 followed by NIR light irradiation compared to the control group. (*n* = 10 in each group, ****P < 0.0001: Tra-IR700 and Per-IR700 with NIR light vs. control, **P = 0.0033: Tra-IR700 with NIR light vs. control; long-rank test) (Figure 4B).

## DISCUSSION

Trastuzumab and pertuzumab recognize different epitopes of HER2. In this study, we demonstrated that combination treatment of IR700-conjugated trastuzumab and pertuzumab enhanced HER2-specific IR700 fluorescence accumulation both *in vitro* and *in vivo*, resulting in a strong phototherapeutic effect upon NIR light irradiation under the guidance of IR700 fluorescence. This phototherapeutic effect was far superior to that by treatment with the agents singly and followed by NIR light.

PIT is a highly selective cancer therapy, which is based on a molecularly targeted mAb conjugated to the photosensitizer IR700 and exposure to NIR light. We have reported that HER2 target-specific cell death was induced by only 1 dose of Tra-IR700 and 1 exposure to NIR light. However, some cancer cells survived after PIT, and tumor recurrences were eventually seen in mouse models [11, 19], thus it is necessary to develop a new method for enhancing PIT treatment effect. Given that the combination treatment of these 2 different anti-HER2 mAbs induced a stronger antitumor effect on HER2-expressing cancers than by treatment with the agents alone in preclinical and clinical settings, we hypothesized that the combination treatment of these 2 anti-HER2 mAbs conjugated with IR700 and followed by NIR light irradiation would also lead to a stronger antitumor effect than with either agent and NIR light irradiation.

Previous studies showed that the greater the binding of mAb-IR700s is to the target cells, the stronger are the IR700 signals obtained, resulting in a stronger PIT effect upon NIR light irradiation, as evidenced by cell membrane damage through an increased amount of activated IR700 [18]. As shown in Figure 2D, there was a positive correlation between HER2-specific IR700 signals and NIR light-induced phototoxicity in various types of HER2 expression, which was consistent with the findings of a previous study [18]. However, there is a limit of mAb-IR700 binding to each target cell owing to saturation of antigens. Tra-IR700 and Per-IR700 were capable of binding to HER2 without competing with each other, and the combination treatment of these 2 agents could yield higher IR700 signals that could not be achieved by single-agent treatment owing to its antigens being saturated. Combination treatment of both Tra-IR700 and Per-IR700 followed by NIR light irradiation induced stronger phototoxicity than that with treatment with either single agent followed by NIR light (Figures 1E, 2A, 2C). Therefore, similar to the previous study, which used 2 types of mAbs recognizing different molecules for PIT, combination treatment using 2 types of mAbs recognizing different epitopes of the same molecule enables higher IR700 localization of the target cells, resulting in stronger phototoxic effect than single-agent-mediated PIT [20].

Consistent with *in vitro* studies, quantitative analysis showed an additive increase in HER2-specific IR700 fluorescence in NCI-N87 tumors over time after injection of both 100 μg of Tra-IR700 and 100 μg of Per-IR700 compared to injection of the either single agent (Figure 3A, 3B), and image analysis showed the same localization of trastuzumab and pertuzumab when administrated simultaneously (Figure 3C), demonstrating that Tra-IR700 and Per-IR700 accumulated in the target tumors without competition with each other. Therefore, sufficient and preferable tumor distribution of mAb-IR700 was achieved with the combined use of both types of mAb-IR700, resulting in an enhanced antitumor effect and prolonged survival (Figure 4A, 4B). However, we still found a tumor recurrences even in Tra-IR700- and Per-IR700-mediated PIT group. Tumor microdistribution of mAb is not generally uniform, which may hamper cytotoxic and phototoxic effects of treatment [21]. Previous studies shows that PIT treatment enables better tumor microdistribution of nanosized agents, leading to improve treatment outcomes [22], and that repeated PIT treatment enables better tumormicrodistribution of mAb from peripherally to uniformly leading to better tumor control [23]. These methods should improve the efficacy of Tra-IR700- and Per-IR700-mediated PIT.

Importantly, target-specific IR700 imaging can be used to assess molecular expression in the tumor, cytotoxic effects of mAbs as well as phototoxic effects of mAb-IR700 upon NIR light irradiation, and therapeutic response (remnant viable tumor). By using an endoscope or laparoscope, imaging and phototherapy can be performed under endoscopic and surgical settings.

Target tumor cell-selective therapy was performed without significant adverse effects by PIT; however, in contrast, tumor heterogeneity is a possible limitation of this method. Use of chemotherapeutic agent in addition to PIT or PIT using IR700 conjugated multiple mAbs targeting different molecules could solve this issue [20, 22, 24]; however, further study should be performed such as developing mAb-IR700 plus radioisotope/toxin conjugates. In addition, orthotopic tumor models should be tested for further studies because it is considered more clinically relevant models of therapeutic efficacy than xenograft tumor model [25, 26]. In conclusion, the combination treatment of HER2-expressing cancer cells with IR700-conjugated trastuzumab and pertuzumab enhanced HER2-specific IR700 fluorescence accumulation both *in vitro* and *in vivo*, resulting in a strong phototherapeutic effect upon NIR light irradiation. While conventional photodynamic therapy photosensitizers lack tumor-specificity leading to cause unwanted side effects, mAb-IR700 conjugates enables molecular target-specific fluorescence imaging and phototoxicity upon NIR light irradiation without significant side effects [27–29]. This strategy expands the capability of imaging-guided molecular targeted therapy for HER2 expressing cancers, such as breast and gastric cancer, in enabling better outcomes in cancer patients.

## MATERIALS AND METHODS

### Reagents

Two different anti-HER2 mAbs, trastuzumab (Herceptin^®^) and pertuzumab (Perjeta^®^), were purchased from Chugai Pharmaceutical Co. Ltd. (Tokyo, Japan). IRDye700DX NHS ester (IR700) was purchased from LI-COR Biosciences (Lincoln, NE, USA). Alexa Fluor488 NHS ester (Alexa488) was purchased from (Life Technologies, Gaithersburg, MD, USA).

### Synthesis and purification of IR700-conjugated trastuzumab or pertuzumab and Alexa488-conjugated trastuzumab

Trastuzumab or pertuzumab (1.0 mg, 6.8 nmol) was incubated with IR700 (66.8 μg, 34.2 nmol) in 0.1 M Na_2_HPO_4_ (pH 8.5) at room temperature for 1 h. The mixture was purified with a Sephadex G50 column (PD-10; GE Healthcare, Piscataway, NJ, USA). Trastuzumab was also incubated with Alexa488 (37.1 μg, 65 nmol) and purified in the same manner as above. The concentrations of protein, IR700, and Alexa488 were measured by absorption at 280 nm, 689 nm, and 494 nm, respectively, using spectroscopy (UV-1800; Shimadzu Corp., Kyoto, Japan) to confirm the number of fluorophore molecules conjugated to each mAb molecule. The number of fluorophore molecules per mAb molecule was adjusted to approximately 3 for IR700 and 5 for Alexa488.

### Cell lines and culture conditions

HER2-expressing human gastric cancer NCI-N87 cells, and human breast cancer HCC-1419 and HCC-1954 cells were purchased from American Type Culture Collection (ATCC) (Manassas, VA, USA), and human gastric cancer MKN-45 cells were purchased from the JCRB Cell Bank (Tokyo, Japan). We also used *HER2* gene-transfected NIH/3T3 (3T3/HER2) cells [30]. Parental NIH/3T3 (HER2-negative) cells were purchased from ATCC. Cells were cultured with RPMI 1640 (Life Technologies, Gaithersburg, MD, USA) supplemented with 10% fetal bovine serum and 1% penicillin/streptomycin (Life Technologies) in tissue culture flasks in a humidified incubator at 37°C in an atmosphere of 95% air and 5% carbon dioxide.

### Determination of HER2 expression *in vitro*

To determine HER2 expression of the cells, IR700 fluorescence was measured by flow cytometry analysis (MACSQant analyzer; Miltenyi Biotec, Bergisch Gladbach, Germany) after treatment with Tra-IR700 or Per-IR700. Cells were seeded at 5 × 10^5^/well on 35-mm dishes and incubated for 48 h at 37°C. The medium was replaced with fresh culture medium containing 10 μg/ml of Tra-IR700 or 10 μg/ml of Per-IR700 and incubated for 3 h at 37°C. Cells were washed with PBS, and flow cytometry analysis was performed. To confirm the target specificity of Tra-IR700 or Per-IR700, excess unconjugated trastuzumab or pertuzumab (100 μg) was added to block HER2 molecules before Tra-IR700 or Per-IR700 treatment. The MFIs were evaluated and compared to the MFI of the isotype control. To confirm HER2-specific co-localization of trastuzumab and pertuzumab, cells were seeded at 2 × 10^4^/well on cover glass-bottomed dishes and incubated for 24 h at 37°C. Both Tra-Alexa488 (10 μg/ml) and Per-IR700 (10 μg/ml) were then added to the culture medium and incubated for 3 h at 37°C. Cells were washed with PBS, and fluorescence microscopy was performed (IX73; Olympus, Tokyo, Japan) with the following filter settings: 608–648-nm excitation filter and 672–712-nm emission filter for IR700 and 470–495-nm excitation filter and 510–550-nm emission filter for Alexa488. All fluorescence images were analyzed with ImageJ software (http://rsb.info.nih.gov/ij/).

### Determination of mAb-IR700 dose response relationship of HER2-specific fluorescence intensity *in vitro*

To evaluate the IR700 fluorescence intensities of the cells after treatment with Tra-IR700 plus Per-IR700 or either agent alone, flow cytometry analyses were performed. Cells were seeded at 5 × 10^5^/well on 35-mm dishes and incubated for 48 h at 37°C. The medium was replaced with fresh culture medium and then incubated with 0.1, 1, 10, 100 μg/ml of Tra-IR700; 0.1, 1, 10, 100 μg/ml of Per-IR700; or 0.1, 1, 10, 100 μg/ml each of Tra-IR700 and Per-IR700 for 24 h at 37°C. Cells were washed with PBS, after which flow cytometry analysis was performed and MFIs were determined.

### *In vitro* PIT

Cells were seeded at 5 × 10^5^/well on 35-mm dishes and incubated for 48 h at 37°C. The medium was replaced with fresh culture medium containing Tra-IR700 (1 or 10 μg/ml) plus Per-IR700 (1 or 10 μg/ml), or either agent alone (1 or 10 μg/ml), and incubated for another 24 h at 37°C. Cells were washed with PBS, and phenol red-free RPMI 1640 was added. Then, cells were irradiated with NIR light (1 J/cm^2^ or 2 J/cm^2^) using a light-emitting diode emitting light at 670–710 nm (L690-66-60; Epitex Inc., Kyoto, Japan). Power density of 24 mW/cm^2^ was measured with an optical power meter (PM 100, Thorlabs, Newton, NJ, USA).

### Cytotoxicity assay

The cytotoxic effect in response to PIT was determined by the LIVE/DEAD^®^ Fixable Green Dead Cell Stain Kit (Life Technologies), which can detect damaged cellular membranes by flow cytometry [31]. Cells were trypsinized just after NIR light irradiation and were washed with PBS. LIVE/DEAD green fluorescent reactive dye was added to the cell suspension and incubated for 30 min at room temperature, followed by flow cytometry analyses. To further determine the long-term cytotoxic effects of PIT with both Tra-IR700 and Per-IR700, a trypan blue dye exclusion assay was performed. Cells were seeded at 5 × 10^4^ on 35-mm dishes and incubated for 48 h at 37°C. The medium was replaced with fresh culture medium containing both Tra-IR700 (10 μg/ml) and Per-IR700 (10 μg/ml), or either agent, and incubated for another 24 h at 37°C. Cells were washed with PBS, and phenol red-free RPMI 1640 was added. Then, cells were irradiated with NIR light. At the indicated time point after NIR light irradiation, cells were collected, and viable cells were counted with an automated cell counter (Countess; Life Technologies) based on trypan blue dye uptake.

### Correlation between HER2-specific IR700 fluorescence intensity and phototoxicity in response to PIT

To assess the correlation between HER2-specific IR700 fluorescence intensity from Tra-IR700-treated cells before NIR light irradiation and phototoxicity in response to PIT by using 6 cell lines (NCI-N87, NIH/3T3, HCC-1419, HCC-1954, MKN-45, and 3T3/HER2), IR700 fluorescence intensities were evaluated by a flow cytometer after 24-h incubation with 10 μg/ml of Tra-IR700, and the LIVE/DEAD assay was performed just after NIR light irradiation (24-h incubation with 10 μg/ml of Tra-IR700, followed by 2 J/cm^2^ of NIR light irradiation).

### Xenograft tumor model

Six-week-old female BALB/c-nu/nu mice (CAnN.Cg-Foxn1^nu^/CrlCrlj nu/nu) were obtained from Charles River Laboratories Japan, Inc. (Yokohama, Japan). All mice were allowed to acclimatize and recover from shipping-related stress for 1 week before the studies, and were kept under a controlled light-dark cycle (12:12 h). All animal studies were conducted in accordance with the guidelines established by the Animal Care Committee at the Jikei University School of Medicine. Five million NCI-N87 cells were injected subcutaneously into the right dorsum of the mice. The tumor xenografts were measured with an external caliper, and the tumor volume was calculated using the following formula: length × width × height × 0.5 [32].

### *In vivo* fluorescence imaging

To determine the biodistribution of Tra-IR700 or Per-IR700 and evaluate IR700 fluorescence intensities in the target tumors, fluorescence images were obtained with the IVIS^®^ Imaging System (Caliper Life Sciences, Hopkinton, MA, USA) using a 675-nm excitation filter and a 695–770-nm emission filter. NCI-N87 tumors reaching approximately 20 mm^3^ in volume were selected and randomized into 7 groups of at least 3 mice per group as follows: (i) control (PBS i.v.), (ii) 10 μg of Tra-IR700 i.v., (iii) 10 μg of Per-IR700 i.v., (iv) 10 μg of Tra-IR700 and 10 μg of Per-IR700 i.v., (v) 100 μg of Tra-IR700 i.v., (vi) 100 μg of Per-IR700 i.v., and (vii) 100 μg of Tra-IR700 and 100 μg of Per-IR700 i.v. IR700 fluorescence images were obtained 1, 2, 3, and 5 days after the injection using the same settings of exposure time, camera binning, and stage height under isoflurane anesthesia. All fluorescence images were analyzed with ImageJ software. The regions of interest were manually determined on tumor area depending on where the IR700 fluorescence was localized. Furthermore, to detect the localization of trastuzumab and pertuzumab in the target tumors when administrated simultaneously, both NCI-N87 and NIH/3T3 tumor-bearing mice were created. Mice were injected intravenously with Tra-Alexa488 and Per-IR700. Fluorescence images were obtained 2 days after injection using the same settings as described above. To detect Alexa488 signals, a 465-nm excitation filter and a 515–575-nm emission filter were used.

### PIT *in vivo*

To determine the antitumor effects in response to PIT, the following experiments were conducted. NCI-N87 tumors reaching approximately 20 mm^3^ in volume were selected and randomized into 8 groups of 10 mice per group as follows: (i) control (PBS i.v. without NIR light irradiation), (ii) PBS i.v. followed by NIR light irradiation (100 J/cm^2^) on day 1 after injection, (iii) 100 μg of Tra-IR700 i.v. without NIR light irradiation, (iv) 100 μg of Per-IR700 i.v. without NIR light irradiation, (v) 100 μg each of Tra-IR700 and Per-IR700 i.v. without NIR light irradiation, (vi) 100 μg of Tra-IR700 i.v. followed by NIR light irradiation (100 J/cm^2^) on day 1 after injection, (vii) 100 μg of Per-IR700 i.v. followed by NIR light irradiation (100 J/cm^2^) on day 1 after injection, and (viii) 100 μg each of Tra-IR700 and Per-IR700 i.v. followed by NIR light irradiation (100 J/cm^2^) on day 1 after injection. NIR light irradiation was performed under isoflurane anesthesia with a 690-nm continuous wave laser at a power density of 330 mW/cm^2^ (ML6540-690; Modulight, Inc., Tampere, Finland). After the treatments, tumor volumes were measured thrice a week until the volume reached 500 mm^3^.

### Statistical analyses

Mean ± standard error of the mean (SEM) values from a minimum of 3 experiments were determined. Statistical analyses were carried out using GraphPad Prism software (GraphPad Software Inc., La Jolla, CA, USA). Student's *t*-test was used to compare the 2 treatment groups. Pearson's correlation coefficient was used to analyze the correlation between the IR700 fluorescence intensity and the ratio of dead cells. For *in vivo* experiments, the Mann–Whitney *U* test was used to evaluate the differences in tumor volume between the 2 treatment groups. The cumulative probability of survival was estimated in each treatment group by Kaplan–Meier survival curve analysis, and the results were compared by the log-rank test. *P* < 0.05 was considered to indicate a statistically significant difference.

## SUPPLEMENTARY MATERIAL FIGURES


