# Supplementary material for: Combination photoimmunotherapy with monoclonal antibodies recognizing different epitopes of human epidermal growth factor receptor 2: an assessment of phototherapeutic effect based on fluorescence molecular imaging
Source: Oncotarget. 2016 Feb 19;7(12):14143–52. doi: 10.18632/oncotarget.7490 (PMC4924703; doi:10.18632/oncotarget.7490)
Supplement: Supplementary file 1 [file oncotarget-07-14143-s001.pdf]

Combination photoimmunotherapy with monoclonal antibodies recognizing different epitopes of human epidermal growth factor receptor 2: an assessment of phototherapeutic effect based on fluorescence molecular imaging

Supplementary Materials

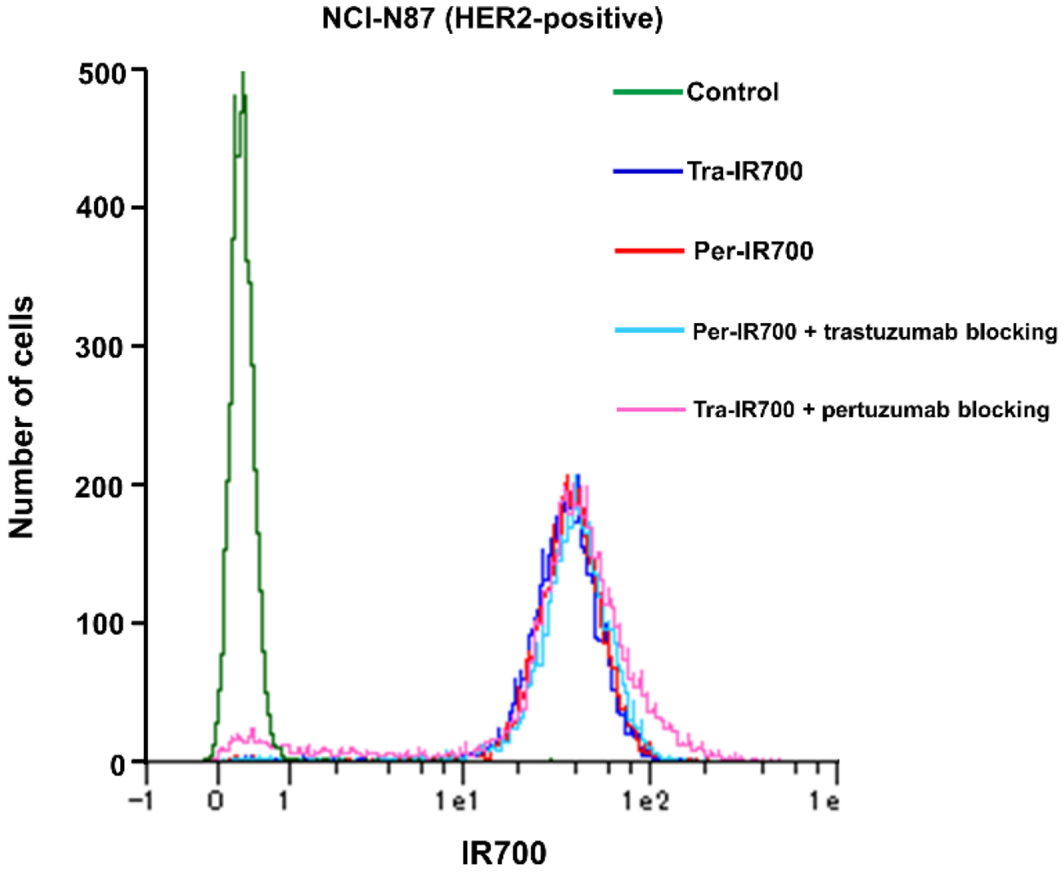

Supplementary Figure S1: Flow cytometry analysis. Tra-IR700 binding was not blocked by excess unconjugated pertuzumab, and Per-IR700 binding was not blocked by excess unconjugated trastuzumab in HER2-expessing NCI-N87 cells.

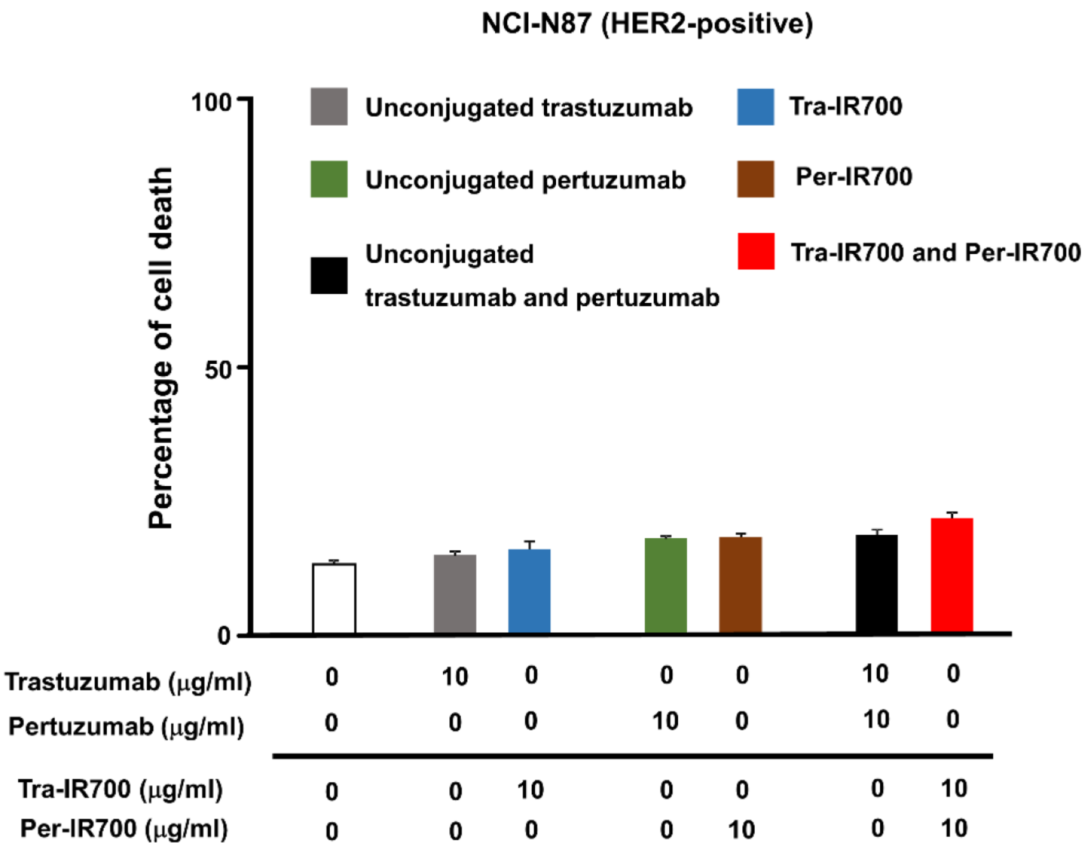

Supplementary Figure S2: LIVE/DEAD assay. There was no significant difference in cytotoxicity between trastuzumab and Tra-IR700, between pertuzumab and Per-IR700, or between the combination of trastuzumab and pertuzumab and combination of Tra-IR700 and Per-IR700 treatment in NCI-N87 cells. Data are presented as means ± SEM (n = 3, Student's t test).

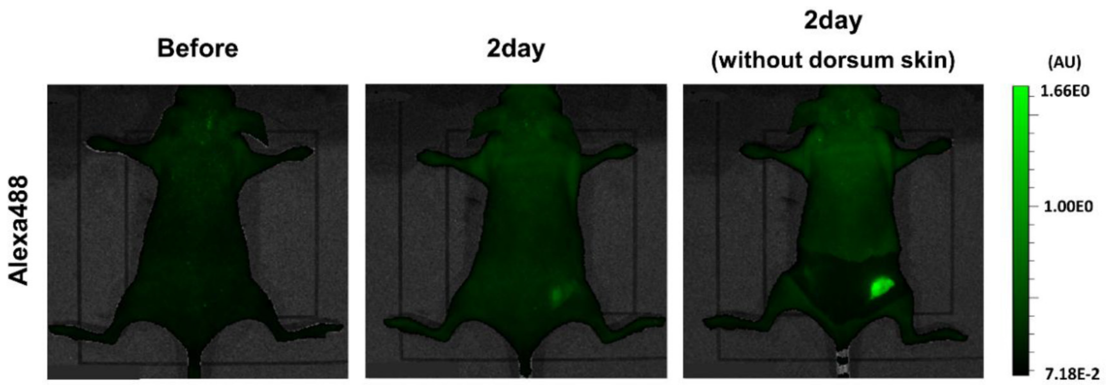

Supplementary Figure S3: NCI-N87 (right dorsum) and NIH/3T3 (left dorsum) tumor-bearing mouse models. Alexa488 fluorescence was clearly detected in NCI-N87 tumors when the dorsum skin was removed (2 days after an injection of 50 μg Tra-Alexa488).
